# Cadmium-Based Quantum Dots Alloyed Structures: Synthesis, Properties, and Applications

**DOI:** 10.3390/ma16175877

**Published:** 2023-08-28

**Authors:** Fadia Ebrahim, Omar Al-Hartomy, S. Wageh

**Affiliations:** 1 Department of Physics, Faculty of Science, King Abdulaziz University, Jeddah 21589, Saudi Arabia; fabdullahebrahim@stu.kau.edu.sa (F.E.); oalhartomy@kau.edu.sa (O.A.-H.); 2 Department of Physics, College of Applied Science, Umm Al-Qura University, Makkah 21955, Saudi Arabia; 3 K. A. CARE Energy Research and Innovation Center, King Abdulaziz University, Jeddah 21589, Saudi Arabia; 4 Physics and Engineering Mathematics Department, Faculty of Electronic Engineering, Menoufia University, Menouf 32952, Egypt

**Keywords:** alloyed ternary quaternary quantum dots, II–VI compounds, cadmium chalcogenides

## Abstract

Cadmium-based alloyed quantum dots are one of the most popular metal chalcogenides in both the industrial and research fields owing to their extraordinary optical and electronic properties that can be manipulated by varying the compositional ratio in addition to size control. This report aims to cover the main information concerning the synthesis techniques, properties, and applications of Cd-based alloyed quantum dots. It provides a comprehensive overview of the most common synthesis methods for these QDs, which include hot injection, co-precipitation, successive ionic layer adsorption and reaction, hydrothermal, and microwave-assisted synthesis methods. This detailed literature highlights the optical and structural properties of both ternary and quaternary quantum dots. Also, this review provides the high-potential applications of various alloyed quantum dots.

## 1. Introduction

Colloidal semiconductor nanocrystals, or quantum dots (QDs), are powerful tools of great importance in many applications because of their small nanoscale size, which is smaller than the Bohr exciton radius (~1–20 nm) [1,2,3,4,5]. The main advantage of this size reduction is presented in the major changes that occur to the material properties compared to the bulk. One of the most important reasons behind these changes is the quantum confinement that appears when the size is reduced until it becomes smaller than Bohr’s radius, which limits the degree of freedom of the charge carriers by making them confined in the material’s nanoscale dimension. Depending on their confinement, nanomaterials can be classified into 2-dimension (2D), 1-dimension (1D), and 0-dimension (0D). Quantum dots belong to the latter type, where the charge carriers are confined in the three dimensions, leading to drastic variations in the electronic states and, accordingly, the band structure, which becomes discrete in the case of quantum dots rather than the continuous band structure of the bulk material [6], which was found to cause a shift of the absorption maximum to lower wavelengths (blue range). The blue shift that occurs with decreasing the size is evidence of the high band gap due to the inverse proportionality between the size and the band gap [4,7,8]. The band gap is not the only property that is altered but also the ability of the nanomaterial to allow/prevent charge carriers from flowing through it, which is expressed by conductivity [4]. Another feature that appears with nano-size materials is the high surface-to-volume ratio, and this high ratio creates more places for the surface state, making the surface of the material so active since they have dangling bonds on the surface and hence giving the quantum dots the chance to agglomerate to produce larger particles, which makes them less stable [7,9].

In the recent past, QDs have attracted attention for a wide range of technological applications such as solar cells [10,11,12], light-emitting diodes (LEDs) [13], and displays [14], in addition to various biological applications [15]. This is due to their unique electrical and optical properties, which can be tuned with size control [5], like the wide absorption and sharp emission bands [16,17,18]. Among different types of colloidal nanocrystals, binary Cd-based quantum dots such as CdSe, CdS, and CdTe are considered one of the most commonly used QDs since their fluorescence shows excellent properties that can be observed in a wide range of the optical spectrum [19]. In spite of the excellent optical properties of binary QDs, the size dependence of the optical and electronic properties can be a limitation for binary QDs. So, the capping is applied to these nanocrystals to prevent agglomeration and maintain their small size, but it can affect their luminescent performance and lower their quantum efficiency [20,21,22,23]. However, alloyed QDs such as ternary CdSeTe [12,24] and quaternary CdSeTeS and CdZnSeS [25,26,27] have shown superior properties that can be controlled by managing the composition of the reactants even without changing the size, which is a good property for some applications that require a specific size [26,27,28]. Due to the increasing demand for alloyed Cd-based quantum dots, more research is required to provide more information about these nanoparticles. In this study, we report the synthesis techniques for Cd-based QDs in addition to their optical and structural properties and their applications.

## 2. Materials and Methods

Many researchers have developed different preparation techniques for alloyed ternary and quaternary quantum dots. Hot injection [25], the colloidal method in an aqueous medium [7], successive ionic layer adsorption and reaction (SILAR) [29], hydrothermal [12], and microwave-assisted [30] are the most commonly used methods for the preparation of cadmium chalcogenide nanostructures. The main idea of the synthesis of alloyed nanocrystals (NCs) is to grow the ternary or quaternary alloy on the base of a binary seed [19].

### 2.1. Hot-Injection

The hot injection technique has been commonly used to synthesize various alloyed nanomaterials, in particular cadmium chalcogenides, including; CdSeS [19], CdZnS [31], CdSeTe [28], and CdSeSTe [25]. This technique was introduced by Murray et al. [32] in 1993 for the first time. In 2015, Adejoke et al. prepared CdSeS/ZnSeTe core/shell QDs; they started with the preparation of chalcogen anionic precursor (Te precursor) by mixing tellurium powder with trioctylphosphine oxide (TOPO). Then, they poured it into the metallic cationic precursor (Cd precursor), which was obtained by mixing cadmium oxide with powder, 1-octadecene (ODE), and oleic acid (OA). Before adding the tellurium precursor, the cadmium precursor was preheated to 280 °C with the flowing argon; in this way, the binary compound was generated. After that, they injected the sulfur precursor to obtain the ternary alloyed CdSeS quantum dot. This was followed by the formation of a ZnSeTe shell to passivate the surface of the CdSeS core, and the temperature was decreased to 200 °C to suppress the growth of the CdSeS. Finally, they rinsed the samples with methanol and acetone [19] to purify the resultant QDs by making them free from any excess elements or solvents, which is an important step, especially when preparing samples for high-resolution transmission electron microscopy (HR-TEM) [33,34]. The size of the CdSeS core was found to be around 2.8 nm, and for the core/shell sample, the size was 3.3 nm. Furthermore, ternary HgCdTe QDs were prepared by hot injection for photo-detector applications with a size of approximately 14 nm, and it was found that the temperature of the reaction, compositional ratio, and growth time are important factors that must be taken into consideration to control the size of the prepared QDs [35].

**Figure 1:**
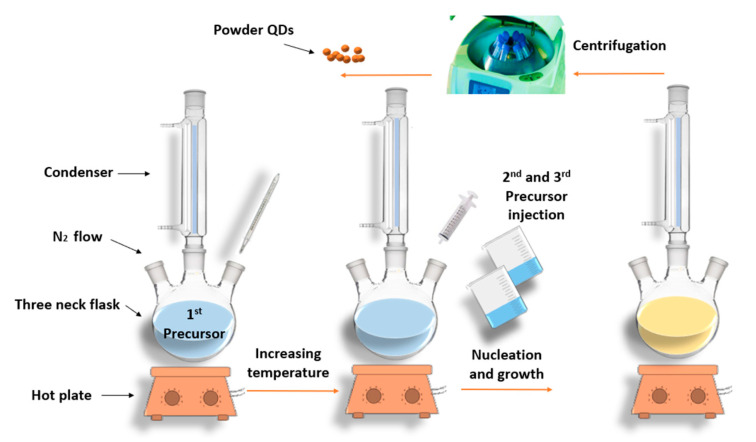
Graphical representation of Q.D. hot-injection synthesis.

Based on different reports [19,36], hot-injection synthesis can be summarized in the following steps. First, all the needed elements should be prepared separately by dissolving each element source in the appropriate solvent. Then, to form the binary seed, the first element source (usually metallic) is injected into the three-neck flask containing the solvent, which is kept under magnetic stirring on the hot plate [4]. Followed by raising the temperature. After that, the second element is introduced to the same flask, and here is where the binary seed is created. Finally, to establish the nucleation and growth of the ternary alloy, the third element solution is added [19,25]. The process takes place under the flow of an inert gas (commonly argon or nitrogen) to avoid oxidation and degas the reaction flask [37]. A water condenser is used to cool down the components of the system during the refluxing process [4]. To inhibit the overgrowth of the formed quantum dots, their active surface can be passivated by an appropriate shell at a lower temperature. The next step is obtaining the precipitates from the crude solution by exposing the mixture to the centrifuging system. To have pure samples and to deactivate their surface by avoiding the free ligands [37], QDs are cleaned from impurities many times by adding water, ethanol, acetone, or methanol drop by drop, depending on the material [25,38]. Finally, the obtained samples are kept in a desiccator to dry and get the powder [36]. Sometimes, a non-solvent is added to the crude solution before centrifuging to assist the size-selective precipitation stage [39]. The hot injection is established with the help of various apparatuses, as shown in Figure 1.

Despite the widespread use of this method, it is not easy to obtain large quantities via this technique since it is difficult to maintain the reaction at a specific temperature during the injection process [40].

### 2.2. Colloidal Method in Aqueous Media (Co-Precipitation)

Co-precipitation is a common wet chemical method to obtain binary and ternary II-VI quantum-confined structures. Through this technique, it is easier to manipulate the preferred molar ratios of the elements in the prepared alloy. In 2007, CdZnS was synthesized by R. Sethi et al. [7], and the size of the obtained nanoparticles was around 3 to 4 nm. Precursor solutions of reaction elements are prepared in the same way as explained in hot injection. First, the inorganic metal salt (acetate, sulfate, etc.) is introduced into the three-neck flask that contains a solvent such as deionized water under stirring at room temperature, followed by the addition of a stabilizing or capping agent (surfactant); the reaction is degassed using nitrogen or argon. Then, the pH of the mixture is adjusted until it gets clear and transparent, which usually happens when the pH value gets close to seven. The reason for pH adjustment is to control the stability of the prepared nanocrystals through the variation of the type of coordination of surfactant with the nanocore and improve their quantum yield [41,42]. After that, the second precursor is added for the formation of the binary QDs, and this addition occurs before raising the temperature, which is one of the differences from the hot-injection strategy. Next, the third precursor is introduced into the mixture in the flask, followed by the reducing agent if needed [43]. Then, the temperature is increased to the required temperature for the specific time. The obtained samples are then centrifuged, followed by washing the obtained products and drying them [7,38]. In [7], the addition of capping was delayed until all precursors were introduced in the three-neck flask. 

**Figure 2:**
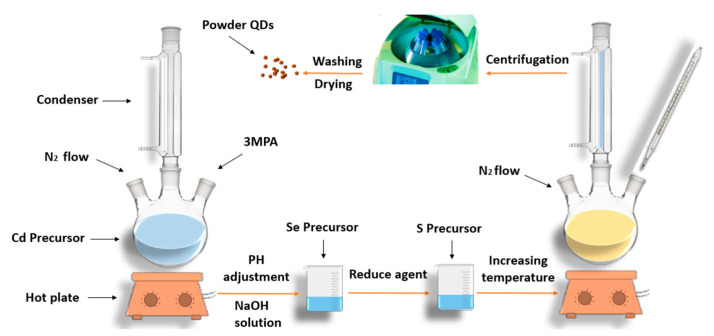
Schematic representation of co-precipitation synthesis.

Co-precipitation was the technique used by many researchers to obtain binary II-VI QDs such as CdSe [37] and CdTe [44], in addition to ternary QDs such as CdZnS [7]. In 2018, S. Wageh et al. synthesized alloyed CdSeS quantum dots through co-precipitation with different selenide/sulfur ratios (Figure 2), and the prepared samples were found to have high crystallinity with a spherical shape, and their size ranged from 2.37 to 3.80 nm [38]. Among different synthesis methods, colloidal is considered a beneficial technique since it is economical and does not require high temperatures or complicated apparatus to establish the process. Moreover, good quantities that are distributed uniformly can be obtained using this method in easy operation conditions that lead to the formation of nanoparticles with constant size, but there is a drawback related to the materials used in this technique. These materials should have relatively similar abilities to solubilize (or dissolve) and precipitate.

### 2.3. Successive Ionic Layer Adsorption and Reaction (SILAR)

**Figure 3:**
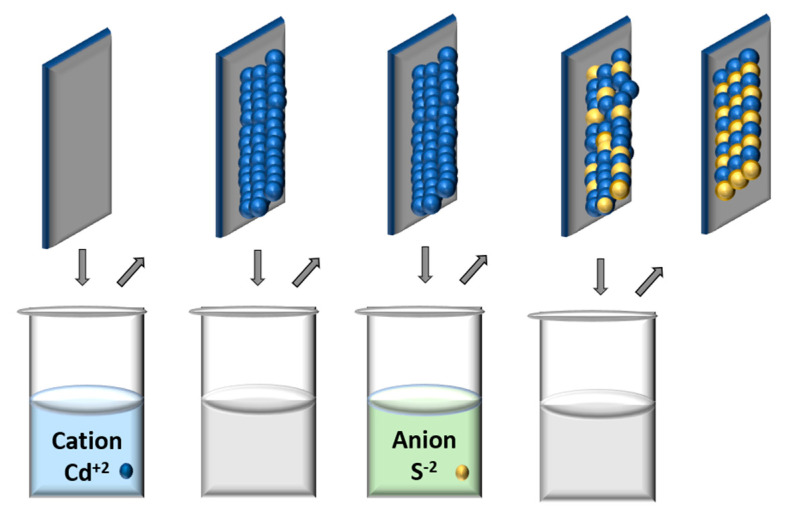
Scheme of SILAR synthesis.

Successive ionic layer adsorption and reaction, or chemical bath decomposition (CBD), is one of the popular chemical deposition techniques used to synthesize thin films. This easy deposition method provides many advantages over the others since it offers the ability to manage the rate of deposition and the molar fractions of the sample components. Moreover, there is no need for vacuum or high temperatures, and it can be done with simple, economical components [45]. Recently, this method has been carried out to prepare quantum dots that are applied in optoelectronics, in particular quantum dot sensitized solar cells (QDSSCs) [11]. In 2020, Omar E. Solis et al. deposited ternary alloyed CdZnS QDs on a substrate by the SILAR method. As described in their experiment, one cycle of the SILAR method started with immersing the substrate in the metal precursor for one minute; the precursor was cadmium acetate dihydrate. The substrate after that was washed using ethanol, followed by repeating the process with the second and third precursors, dehydrated zinc acetate and sodium sulfide, respectively. Finally, a solution of methanol mixed with water was used for washing [46]. In this way, the chemical reaction occurs between the first deposited materials (cationic sources) and the next (anionic sources), so they can combine to introduce the required compound (Figure 3). In 2021, Tyagi and co-authors [10] fabricated CdSSe QDs on TiO_2_/Zn substrates with sizes starting from 3.362 to 10.14 nm, depending on the compositional ratio of selenium and sulfide Se/S.

### 2.4. Hydrothermal

**Figure 4:**
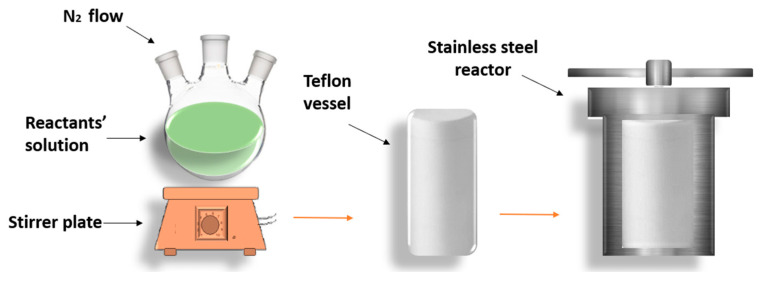
Hydrothermal synthesis of QDs.

Hydrothermal is considered a simple and popular method to obtain various types of nanomaterials, including nanotubes, nanowires [47], nanoparticles [48,49], and nanorods [50]. In 2020 [12], Xiaohui Song’s group synthesized CdSeTe quantum dots that were used for the fabrication of quantum dots-sensitized solar cells (QDSSC); their experiment was established by preparing all the precursors of Cd, Se, and Te separately. The TGA capping (or surfactant) was then introduced to the solution with continuous stirring. After that, sodium hydroxide was added until the pH reached 11.2. The transparent mixture was poured inside the three-neck flask and stirred under nitrogen for twenty minutes. That step was followed by the injection of selenium and tellurium precursors. The solution was poured into an autoclave reactor that was coated with Teflon, and the TiO_2_ substrates were simultaneously put inside. Next, the reactor was sealed and exposed to 160 °C for twelve hours inside the oven, then heated to a higher temperature to strengthen the bond between the QDs and the substrate. Finally, to obtain precipitates, isopropanol was added, followed by centrifuging and drying. By managing the precursors and the conditions of hydrothermal heating, both the shape and structural properties of the samples can be controlled. This method saves energy, material, and money, but the drawback is that it requires a high-cost autoclave and high-temperature operation. The growth of prepared samples cannot be investigated since the reaction is done inside the oven at high temperatures, which is another drawback [51]. A schematic representation of the hydrothermal method is illustrated in Figure 4.

### 2.5. Microwave-Assisted (MW-Assisted)

Microwave-assisted (MW-assisted) synthesis is a technique that is widely used to synthesize quantum dots owing to several benefits. The main advantage is that the components can interact indirectly with microwave irradiation, which leads to heating the system uniformly and hence reduces any effects that may occur from temperature gradients when using other traditional ways to heat the system [52,53], and it produces quantum dots that are well crystalline when the heating rates exceed 100 °C [27]. This method provides the ability to control the amount of applied energy, which helps manage the volumetric and internal heating of the components [54]. In addition, the MW-assisted technique is a time-saving approach since it can produce QDs with a high quantum yield in less than 2.5 h [27], and it is considered a fast, cost-effective, safe, and easy method, which makes it so popular in many technological and research fields [53].

**Figure 5:**
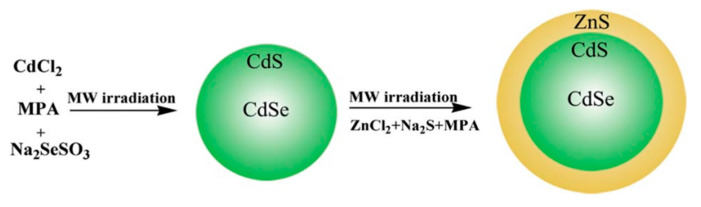
Microwave-assisted preparation of CdSeS/ZnS gradient alloyed core/shell [30].

Many alloyed Cd-based quantum dots have been prepared through the MW-assisted method, including CdWSe and CdSeTe-CdS [55,56]. H.-J. Zhan et al. [30] have synthesized CdSeS gradient-alloyed quantum dots. The gradient alloy consists of CdSe with a thick shell of CdS, and as a source of selenium, they used Na_2_SeSO_3_ instead of other sources like NaHse because that source does not make oxidization reactions easily owing to its good stability in the surrounding environment. In their experiment, they prepared CdSeS as follows: Initially, the monomers from the Cd precursor were allowed to interact with the ions from the Se source while the temperature was low to produce CdSe. After that, the interaction took place between the excess Cd atoms and S ions from the mercaptopropionic acid (MPA) at a high temperature (130 °C) to deposit a thick CdS shell on the outer part of CdSe, forming CdSeS quantum dots after being exposed to microwave irradiation. Finally, the gradient-alloyed QD was covered with a ZnS shell, as illustrated in Figure 5. The size obtained for CdSeS was 2.1 ± 0.6, while after passivation with ZnS, it increased to 3.1 ± 0.9 nm.

**Figure 6:**
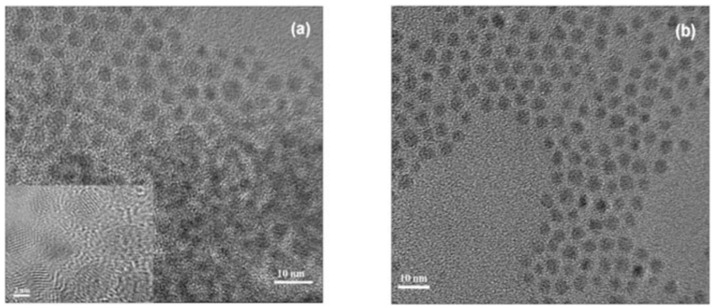
High-resolution Transmission electron microscopy (H-TEM) images of CdSe_x_Te_1−x_S quantum dots after 50 min of reaction: (**a**) x = 0.8; (**b**) x = 0.2 [27].

By the same means, quaternary CdSeTeS was synthesized, and the reactant solutions were prepared in a container made of Teflon from the inside and covered with a very strong Ultem® polyetherimide from the outside before microwave heating. The study revealed that both concentration and the time of heating had considerable effects on the properties. In addition, the use of microwave rays led to the production of high-quality images (Figure 6) of transmission electron microscopy (TEM) owing to the heating method and the interaction between the electromagnetic field from MW irradiation and the reagents [27,53]. In Table 1, The advantages and disadvantages of the mentioned synthesis techniques are illustrated.

## 3. Properties

In the last two decades, high demand has arisen for alloyed quantum dots for potential technological and biological applications like light-emitting diodes [13,67], solar cells, and biolabeling [12,55], which makes it essential to achieve a better understanding of the structural and optical properties of these types of nanostructures to determine their ability for various applications.

### 3.1. Optical Properties

**Figure 7:**
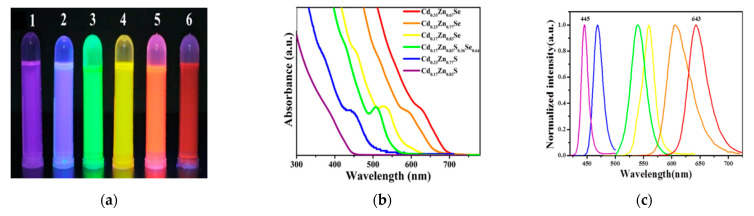
The optical behavior of Cd_x_Zn_1−x_SySe_1−y_ quantum dots with different compositions. (**a**–**c**): (**a**) Samples illuminated by U.V. light; (**b**) UV-vis spectra of the Q.D.s; (**c**) P.L. intensities of the Q.D.s [36].

Cd-based binary quantum dots have been considered one of the most popular semiconductor nanoparticles; they have shown unique optical features that depend on their size [68]. However, binary quantum dots such as cadmium selenide have some limitations, but alloyed QDs can overcome these limitations by managing their optical properties, which can be affected by many factors; the composition of the constituent elements of alloyed samples is one of these factors. In the case of a substitution of Cd by Zn, like CdZnS, the absorption tends to move to a shorter wavelength as the percentage of zinc increases [7]. Furthermore, it was noted that varying the elemental compositions (x and y) of chalcogens and metals in quaternary Cd_x_Zn_1−x_S_y_Se_1−y_ can result in a variety of absorption and emission colors while the size is unchanged, as shown in Figure 7 [36], where a Stokes shift can be seen by comparing the emission peaks to the absorption peaks. This shift between the excitation and emission peaks is important since it describes the features of electronic transitions that occurred between the excitation and emission processes, which affect the optical properties [69]. It also expresses the electron–hole interaction that occurs after the electrons get excited, relax, and finally move to the ground state, resulting in the emission. High values of Stokes shift are of great importance, especially in light-emitting diodes, since they influence decreasing the fluorescence re-absorption that takes place when the emitted photons are absorbed again by the same quantum dot, which reduces the QY. So, a large Stokes shift has a crucial role in maintaining the QY the same even with high levels of quantum dots on the LED thin film [70].

**Figure 8:**
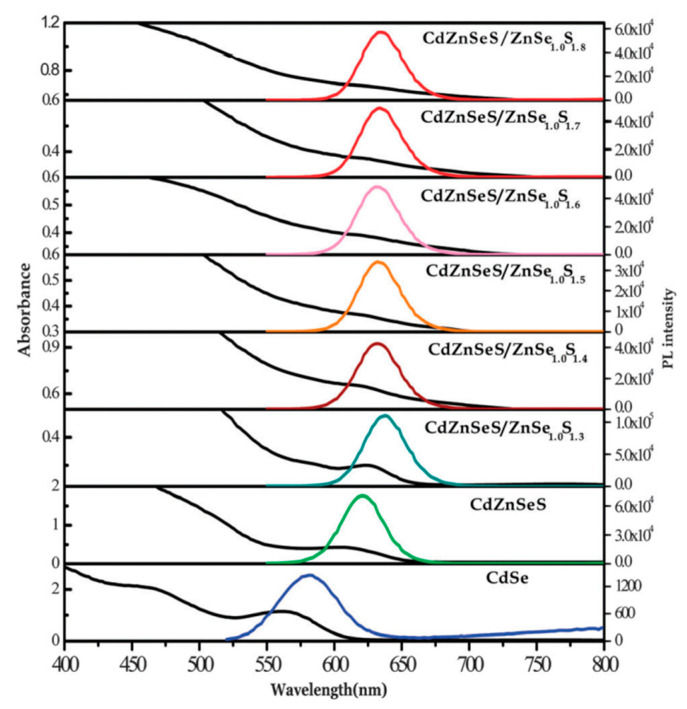
UV-Vis spectra and P.L. intensities of binary CdSe, alloyed CdZnSeS, and core/shell Q.D.s [26].

In addition, the passivation applied to the surface quaternary quantum dot by ternary structures revealed an influence on the optical properties and the noticeable shift of the alloyed spectra, as detected by Adegok et al. [26] and illustrated in Figure 8. On the other hand, coating with multishell causes a shift of absorption to lower energies followed by a shift to higher energies by introducing CdZnS and ZnS shells, respectively. These findings give a strong indication of the change that occurs to the bandgap produced by alloy formation rather than a core/shell model [71], which was confirmed by S. Wageh et al. for CdZnS QDs. It was found that the bandgap of the alloyed Cd_x_Zn_1−x_S QD increased from 3.75 eV to 4.21 eV with increasing Zn concentration [72].

**Figure 9:**
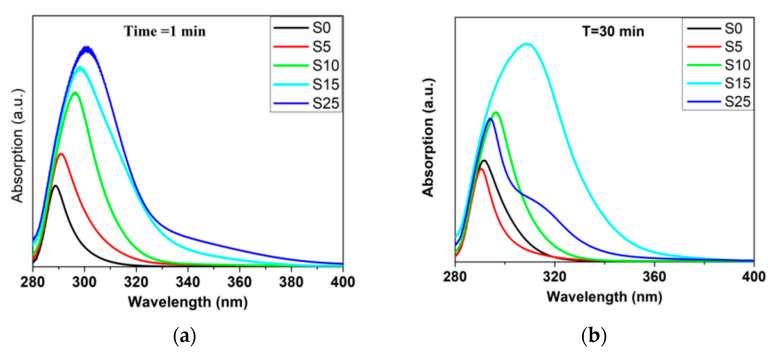

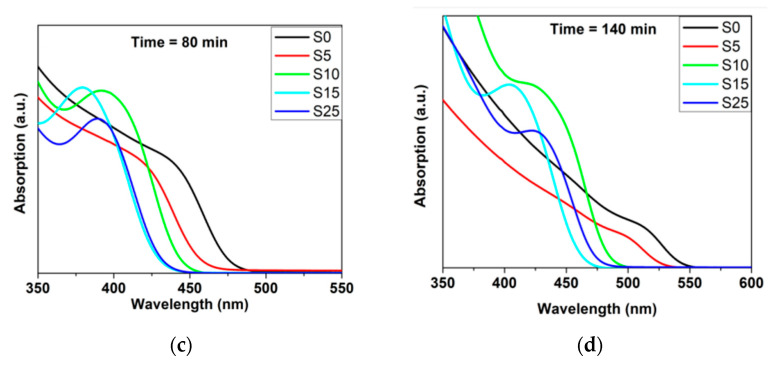
Uv-vis spectra of CdSe and CdSeS during growth with different ratios of S at different times of reaction (t): (**a**) t = 1 min; (**b**) t = 30 min; (**c**) t = 80 min; (**d**) t = 140 min [38].

Similarly, it was reported that varying the ratio of sulfur in CdSeS affects growth since it causes a shift in absorption and emission spectra, as shown in Figure 9. The absorption spectra of these samples were studied through UV-vis spectroscopy at different reaction times, and the influence of composition variation on growth was remarkable, especially after the first minute of reaction (Figure 9a). It can be seen that the absorption curves undergo a red shift with increasing sulfur, and a long extension at the absorption end was observed, which may be because of many reasons, including the difference between the portability of S and Se for chemical reactions and hence in their electronic properties, in addition to the fact that selenium’s atomic radius (2.24 Å) is larger than Sulphur’s (2.14 Å), which leads to a longer bond between Cd and S. The other reason is that the charges of selenium bonds with cadmium are distributed differently from the charges of sulfur bonds with cadmium due to the high electronegativity of S compared to Se. All these reasons led to the creation of vacancies and interstitial defects in the quantum dots [38,73].

Quantum yield, which is defined as the ratio between emission and absorption intensities, is an important parameter that provides a good understanding of quantum dots’ properties. In 2019, Osman’s group studied Cd_x_Zn_1−x_SSe_1−y_; they reported that the quantum yield dramatically increased from 38% to 56% by raising the cadmium ratio, and this value jumped up to 89% when selenium was added to transform to the quaternary Cd_0.17_Zn_0.83_S_0.36_Se_0.64_ [36], and another high QY value of ~98% was recorded in 2022 for CdSeZnS QDs [15]. Adegoke et al. have studied the optical properties of both binary CdSe, pure CdSeS, CdSeS/ZnSeTe, and CdSeS/ZnSeTe/ZnS passivated QDs. It was found that the quantum yield was dramatically improved by 69.5% when moving from CdSe samples to ternary CdSeS, which can be attributed to the reduction of defect states and the decrease of non-radiative recombination. The reduction in surface defects can be achieved through surface passivation, along with improving the ordering of structure due to reaction rates and heteroepitaxial growth [19].

In addition to compositional ratio, other factors have remarkable effects on the optical properties, such as the type of stabilizer used while preparing [37], and it was found that stabilizers (in particular, thiol groups like mercaptosuccinic acid) can easily be used to manage the optical properties [74] since they have a direct effect on the ability of the quantum dots to bond to other components owing to their specific type of bonds with effective properties, which are thiol (–SH) groups and carboxyl (–COOH) functional groups, which can control the availability of chemical reactions on the surface of quantum dots [37,75].

**Figure 10:**
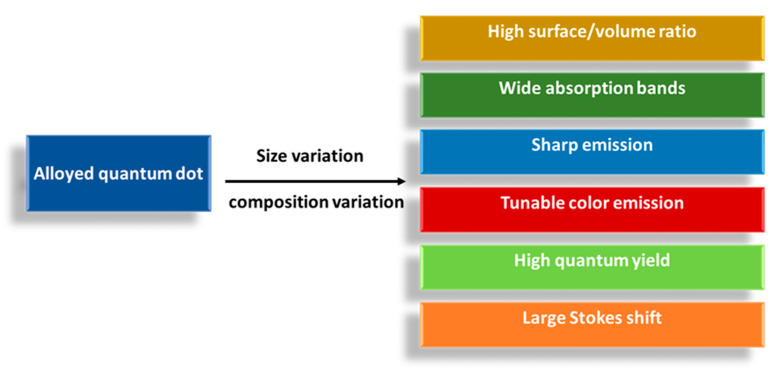
The size and composition dependent optical properties of alloyed quantum dots.

Time of reaction [13,33] is another factor, as shown in Figure 9; even the ability of the nanomaterial to dissolve in water (hydrophilicity and hydrophobicity) [25] can contribute to optical properties. According to all previous researchers, it is very clear that both ternary and quaternary alloyed quantum dots have size/composition-dependent properties, which are illustrated in Figure 10.

### 3.2. Structural Properties

One of the strongest tools to investigate the structural properties of nanoparticles is X-ray diffraction (XRD). Through this technique, structure (cubic, hexagonal, etc.), crystallinity, and even size can be obtained by analyzing the diffraction patterns and using the Debye–Scherrer formula for size calculations.

D = 0.9λ/βcosθ,(1)

D represents the diameter of the nanoparticle, λ represents the wavelength of X-ray radiation, β represents the full-width half at maximum (FWHM) of the peak in radians, and θ represents Bragg angle [75].

**Figure 11:**
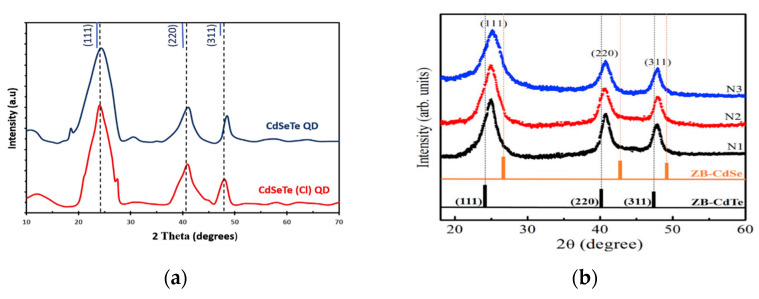
XRD spectra of (**a**) CdSeTe [33]; (**b**) Cd_1−x_Cu_x_SeTe, N1 (x = 0), N2 (x = 0.005), N3 (x = 0.05) [76].

Many efforts have been made to determine the structural features of alloyed II-VI quantum dots based on cadmium. In 2020, both CdSeTe [33] and CdTeSe [76] were studied separately, and their diffraction lines were found to be (111), (220), and (311), which lie between the lines of their parent binary CdSe and CdTe with a zinc blend structure as presented in Figure 11 (a&b(N1 sample)).

**Figure 12:**
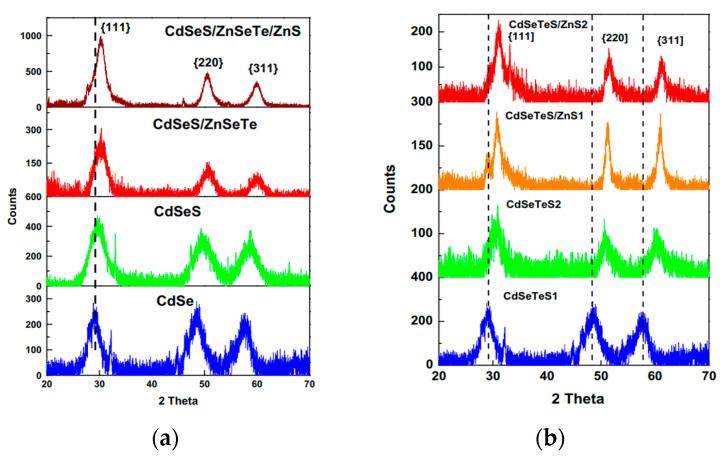
XRD spectra of: (**a**) CdSeS [19]; (**b**) Cd_1−x_Cu_x_SeTe, N1(x = 0), N2(x = 0.005), N3 (x = 0.05) [25].

The existence of diffraction peaks of the alloyed ternary or quaternary QDs in positions that are shifted from the parent peaks gives strong evidence of alloy formation, as revealed by Adegoke’s group in 2015 for CdSeS and CdSeTeS ternary and quaternary samples, respectively [19,25] and by other researchers in their study on CdZnSeS [26], as shown in Figure 12. Both the crystal structure and diffraction planes of different QDs are illustrated in Table 2.

**Figure 13:**
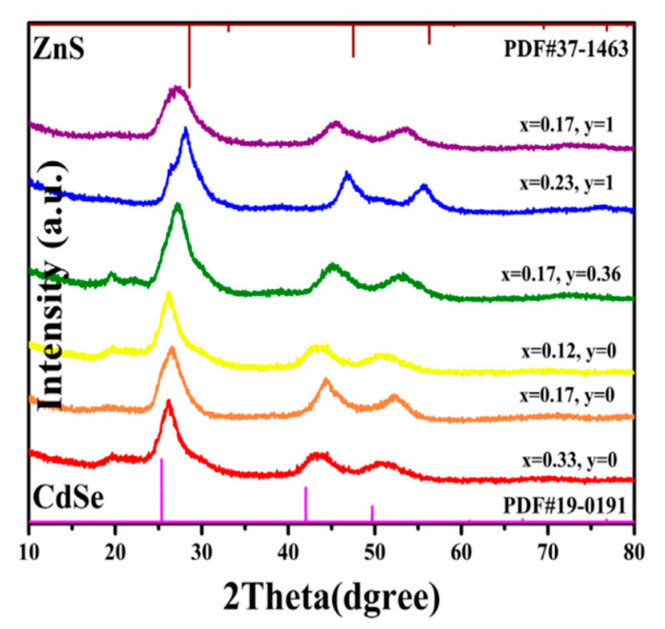
XRD spectra of Cd_x_Zn_1−x_S_y_Se_1−y_ [36].

Furthermore, alloying and the change of the elemental ratios (or composition) have a significant influence on the position of diffraction angles. To illustrate, in quaternary Cd_x_Zn_1−x_S_y_Se_1−y_, the more selenium replaced by sulfur, the greater the shift towards higher diffraction angles (Figure 13); this behaviour leads to a decrease in lattice constant (in accordance with Bragg’s law), which can be attributed to the fact that the ionic radius of the elements Zn, Cd, S, and Se are different [10,26,36]. In addition, it was found that increasing the sulfur concentration in CdSSe increased the size of the quantum dots [10].

Moreover, doping with copper has shown similar effects on XRD patterns in size and lattice parameter calculations of CdTeSe; the size has decreased by 0.5 nm after doping the samples, and the same observation was noticed on lattice parameter values, which decreased from 6.256 to 6.243 Å [76]. In some cases [36], the change of lattice constant with composition follows Vegard’s law, which states that the lattice parameter of an alloy has a linear relationship with the elemental ratio under constant temperature [79].

## 4. Applications

### 4.1. Solar Cells

Recently, solar cells have become in high demand as an energy source that can be renewed, especially with the shortage of fossil fuel supplies. Solar cells (or photovoltaic cells) are devices that are used to absorb optical energy from the sun and transform it into electrical energy. They lie under three categories or generations: the first one is based on a wafer that is commonly made of silicon and can be used as a single or polycrystal structure; this type is known as the first generation of solar cells [11], and the second generation is thin film solar cells; this kind is more economical compared to the first, but it has lower efficiency [80].

**Figure 14:**
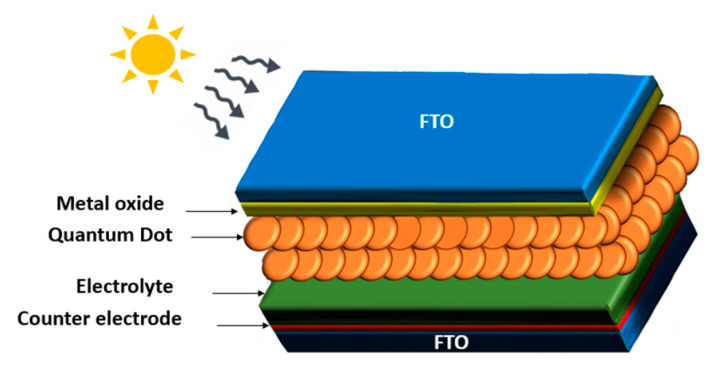
Structure of QDSSCs.

The last type is the third generation, which can be organic, such as dye-sensitized solar cells (DSCSs), or inorganic, based on the sensitizers used, like quantum dot sensitized solar cells (QDSSCs) [81,82]. QD-based photovoltaic cells have been considered competent in solar cell technology owing to their extraordinary properties, like their low, small resistance, multiple exciton generation (MEG) [29], and great optical properties, including the broad absorption range they possess, which can be monitored with their size [34]. It is important to know the structure of QDSCs and how they work to understand quantum dot contributions to them. Basically, they contain three major parts: the photo anode, electrolyte, and counter electrode, as illustrated in Figure 14. These parts are fabricated as follows: quantum dots (as sensitizers or light-absorbing layers) that are placed on a metal oxide that has a large band gap like TiO_2_ or ZnO, and the metal oxide is adjacent to a transparent glass substrate made of a conducting oxide (TCO), usually fluorine-doped tin oxide (FTO), and the collection of quantum dots, metal oxide, and the TCO represents the photo anode [11]. Electrolytes work as a bridge for charges moving between the Q.D. and the counter electrode (C.E.) [81]. Electricity generation follows these steps: (a) When sunlight reaches the Q.D. layer, electrons get excited by photon energy and (b) start moving to the conduction band, leaving holes behind in the valence band (exciton generation); (c) electrons transfer from the C.B. of the quantum dot to the C.B. of the TiO_2_, creating an e-circuit; (d) while holes participate in a redox reaction; (e) the generated photocurrent moves through the conductive glass substrate to the counter electrode [29,83].

Binary Cd-chalcogenides such as CdS, CdSe, and CdTe represent a favorable choice for QDSC fabrication [12,84,85] since they provide a large power conversion efficiency with excellent stability [86]. In recent years, ternary and quaternary Cd-based quantum dots have become more popular relative to binary dots due to their unique optical and electrical properties that depend on their composition instead of size. Thereby, properties like energy band gap can be altered easily only by controlling the elemental ratios of the alloy constituents with constant nano-size, which may give the ability to manage the amount of absorbed sunlight by the solar cell. It was revealed by Song X. et al. that ternary alloyed CdSeTe QDs are more functional compared to their binary parents [12]. For example, solar cells based on ternary alloyed quantum dot absorbers can have conversion efficiencies of up to 8.21% [87]. Mainly, the parameters that describe photovoltaic performance are the short circuit current density (J_sc_, mA/cm^2^), the open circuit voltage (V_oc_, V), the fill factor (FF, %), and the power conversion efficiency (η, %). Both FF and η can be obtained as follows:

(2) $$FF=\frac{P_{max}}{J_{sc}\times V_{oc}}=\frac{J_{max}\times V_{max}}{J_{sc}\times V_{oc}}$$

(3) $$\eta =\frac{P_{max}}{P_{in}}\times 100=\frac{J_{sc}\times V_{oc}\times FF}{P_{in}}\times 100$$

J_max_ and V_max_ represent the maximum values of current density and voltage (V), respectively, P_max_ represents the maximum power out of the device; and P_in_ represents the power of incident light [10]. 

**Figure 15:**
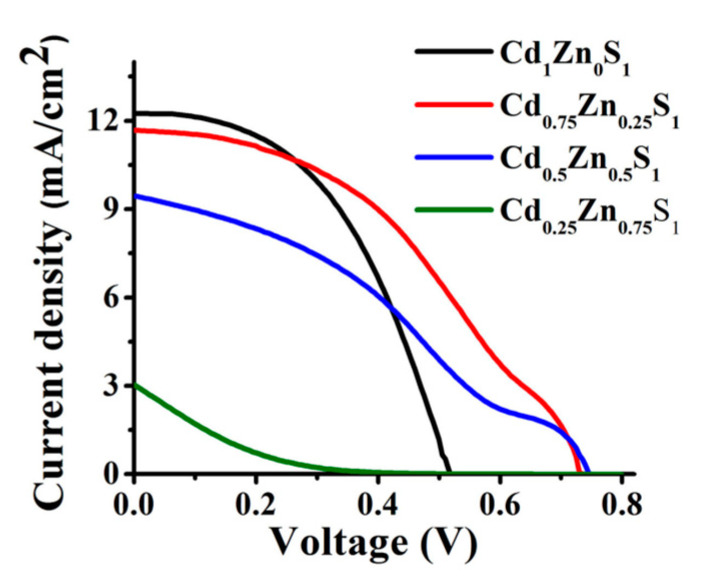
J-V characterization for Cd_x_Zn_1−x_S for x = 0.25, 0.5, 0.75, 1 [46].

The photoelectrical properties of the QDSSCs can be described through current-voltage (J-V) characteristics, which are affected by many factors. Consequently, the photoelectrical properties vary depending on some of the factors, like the elemental ratios of the alloyed quantum dot [12], as revealed by Solis, O.E. et al. [46], who noticed that decreasing the x content in Cd_x_Zn_1−x_S affected J_sc_, η, FF, and V_oc_, while the fill factor decreased with increasing the zinc. In contrast, the open circuit had shown the opposite direction; both the current density and the efficiency had two different behaviors as a response to changing x, as illustrated by Figure 15; the rise in V_oc_ with decreasing x indicates that there may be a change in the alignment of the band and recombination. However, the F.F. was lowered as a result of the existence of energy-active localized defects.

**Figure 16:**
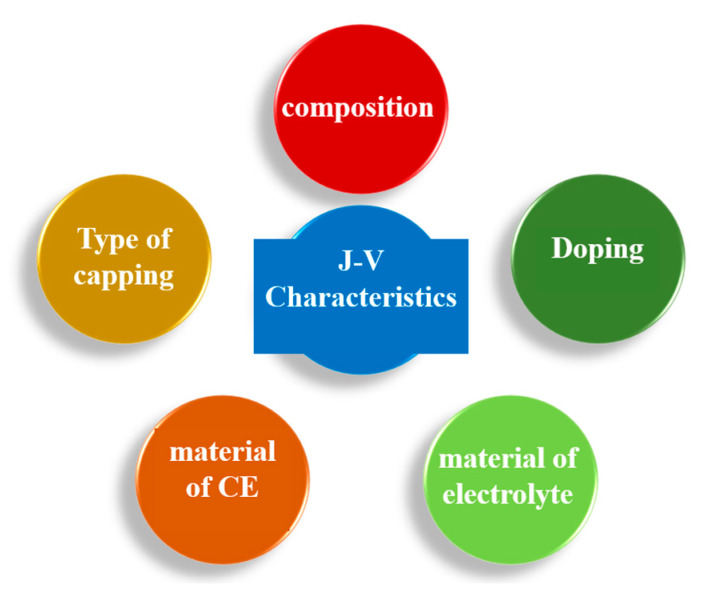
Parameters that affect the J-V characteristics of QDSSCs.

In addition to the compositional ratio, the effects of capping with thiol group agent [12], doping [34], and the type of counter electrode and electrolyte material were all studied by many researchers, as illustrated in Figure 16. Table 3 shows different photovoltaics based on alloyed quantum dots as sensitizers.

### 4.2. Light-Emitting Diodes (LEDs)

Quantum dots are valuable nanostructure materials that represent a great candidate for light emitting diodes (LEDs) [88,89,90,91] because of their optical properties that can be changed with sizes, such as bandgap and large photostability, for core/shell models such as CdTeSe/CdZnS QDs that showed higher stability over UV radiation compared to the binary-based CdTe/CdSe QDs [92]. Additionally, external quantum efficiency (EQE), which represents the numerical percentage between the electrons injected by the device and the emitted light, recorded good values up to 23.68 and 27.6% for CdZnSeS/ZnS and CdZnSeS/ZnS/oleic acid QDs, which can be improved depending on the shell applied [93,94,95,96,97].

Owing to the high activity of QD surfaces, many experiments have applied the passivation shell to the core to decrease the possibility of defect creation and hence manage their properties [98]. Alloyed semiconductors like graded alloyed core/shell CdSeS [99] and CdSeTe [100] quantum dots have been used in LEDs to reduce the surface/volume ratio by varying the band gap through composition change. The alloyed structures are prepared through one-pot techniques or annealed at high temperatures to manage the band gap alignment [94]. In spite of the advantages of the core/shell model, some drawbacks should be taken into consideration, such as thermal decomposition or the oxidation by photons that may occur in the case of using an organic shell and the defects that appear at the core/shell interface like lattice mismatch or dislocations when using inorganic passivation, which affect the quantum efficiency [22,36,90]. Thus, alloyed quantum dots were subjected to interest because of their ability to manipulate their optical properties, like luminescence, by managing the composition, which keeps the nanostructure chemically stable [36]. 

**Figure 17:**
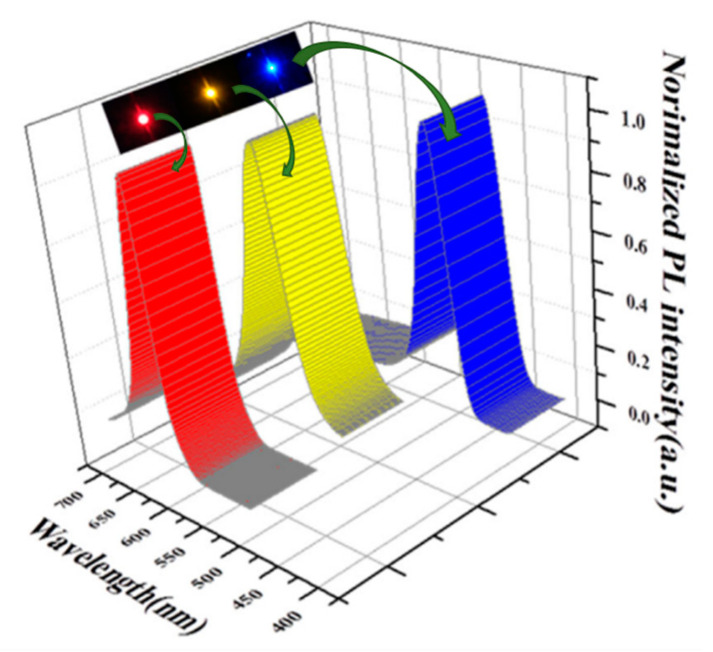
Luminescence spectra of LED based on different compositions of Cd_x_Zn_1−x_S_y_Se_1−y_ QD with V = 3 V [36]. The three compositions are Cd_0.17_Zn_0.83_Se, Cd_0.12_Zn_0.88_Se and Cd_0.23_Zn_0.7_S.

In 2019, Osman H. et al. successively fabricated Cd_x_Zn_1−x_S_y_Se_1−y_ QD on industrial ultraviolet LEDs (365 nm) with three different elemental compositions (x = 0.17, 0.12, 0.23, and y = 0, 0, 1), and the emission spectrum of each device showed a narrow emission band related to each monochromatic colour as shown in Figure 17 [36]. 

**Figure 18:**
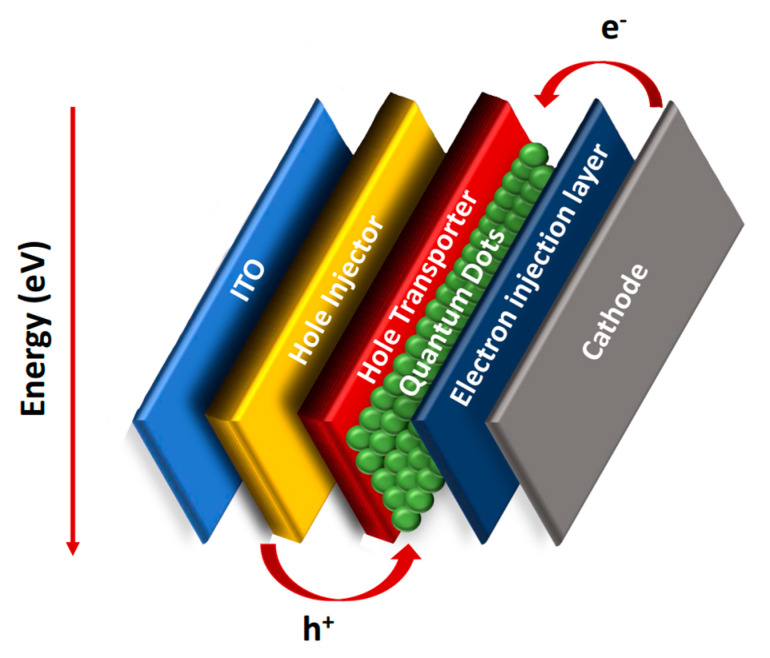
QD-LED and charge transfer.

Furthermore, the QD-based LEDs had excellent stability and emission behavior, which other researchers also observed through their experiment on quaternary CdZnSeS with different molar ratios of the elements. The general device structure starts with a glass substrate coated with indium tin oxide (ITO), followed by a layer that works as a hole injector and, above it, another layer to transport the holes. Then, the quantum dots were applied to this configuration to emit light when exposed to an electric current, which is followed by a layer for the electron injection from the cathode, as shown in Figure 18 [13,36].

### 4.3. Biological Application

The remarkable size-dependent optical properties of QDs have been very encouraging for using them in many biological applications, and they showed promising results, especially in bio-imaging, where they are used as important sensing or detection components for different samples like drugs or food [42,55].

The main idea behind using quantum dots for detection is to observe the effect of the interaction of the compound or material under test on the fluorescence signal of the quantum dots [101]. The advantages of quantum dots, such as their large surface-per-volume ratio, made them promise in chemical sensing. 

In order to prepare QDs for bio-imaging, surface modification should be applied to enhance surface stability, but a reduction in quantum yield was noticed after modification, which is considered a disadvantage in biological uses [102,103,104]. To overcome this, many scientists concentrated on unique alloyed QDs rather than binary-based QDs [78,105,106].

As the biological label is mainly based on the emission of the quantum dots, alloyed quantum dots will be suitable for this application. The alloyed QDs possess the main requirements of biosensors. Alloyed QDS is characterized by tunable luminescence and absorption, high quantum yield, high stability, high resistance to photobleaching, and a large surface-per-volume ratio. In addition, alloying quantum dots could acquire various physical properties with tunable behavior by changing the alloy constituents. The sensing mechanism is based on different photophysical processes. The main processes in quantum dot-based systems are fluorescence energy transfer, charge transfer, and photoinduced electron transfer. The mechanism of fluorescence energy transfer can be followed by a transfer of excitation energy from the donor to the acceptor; the distance between the acceptor and the donor plays an important factor in this mechanism. The charge transfer mechanism can be affected by the variation of the charge distribution in the whole system. The photoinduced electron transfer mechanism can proceed through the gain or loss of electrons by the excited fluorophore, which, in consequence, affects the luminescence intensity. The recent developments in using alloy quantum dots in biological applications will be discussed in the following text [107,108,109].

**Figure 19:**
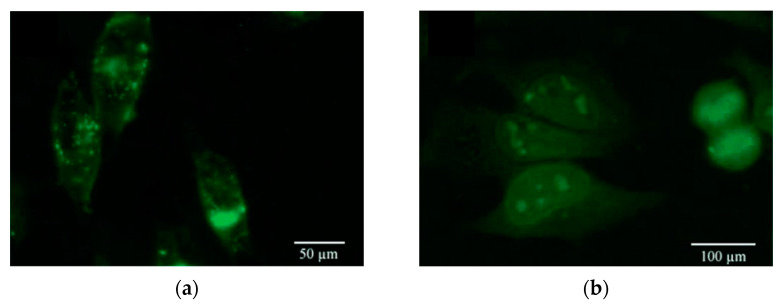
Bio-imaging of Hella cells using CdSeS/ZnS quantum dots: (**a**) one hour after applying Q.D.s.; (**b**) one day after applying Q.D.s [30].

Yang F. Yang F. et al. have used quaternary CdSeTeS bio-probes for cancer cell (SiHa cervical) labeling after applying modification with thiol-PEG-carboxyl (HS-PEG-COOH) and making a coupling or conjugation with Anti-EGFR antibodies. The CdSeTeS bio-probes have shown excellent results in targeted imaging [27]. Furthermore, other researchers reported the synthesis of ternary CdSeS quantum dots (core) passivated with ZnS (shell) for imaging Hella cells, which are living cancer cells. The ZnS shell has made the CdSeS core more stable and, hence, less toxic even after 24 h of interaction with the cells, as shown in Figure 19. In addition, the quantum dots have easily entered the nucleus owing to their small size [30].

**Figure 20:**
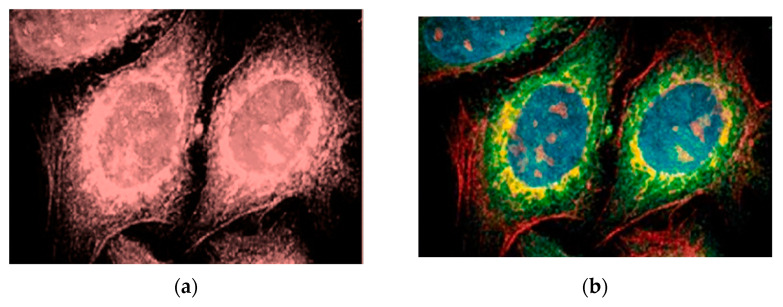
Images of human epithelial cells labeled using: (**a**) organic dye; (**b**) quantum dots [55].

In 2016, alloyed CdZnSeS/ZnSeS quantum dots were employed as highly sensitive biosensors for influenza virus RNA for the first time by the Adegoke, O. group. They prepared different samples of CdZnSeS/ZnSeS quantum dots. Then, they chose the best sample that has an excellent PL quantum yield to make the required bioconjugation. It was found that the biosensor based on alloyed QDs showed extraordinary sensitivity to very low concentrations of the influenza virus H1N1 RNA. In contrast, biosensors based on binary QDs could not detect the virus at such low concentrations [26]. Similarly, highly sensitive electrogenerated chemiluminescence biosensors were fabricated on quaternary CdZnSeS QDs to detect hydrogen peroxide in living cells [110]. Further, El-Hamidy, S., reported that using ternary CdWSe labeling instead of coumarin dye for epithelial cells in humans has a great impact on image quality and disparity, as illustrated in Figure 20 [55].

**Figure 21:**
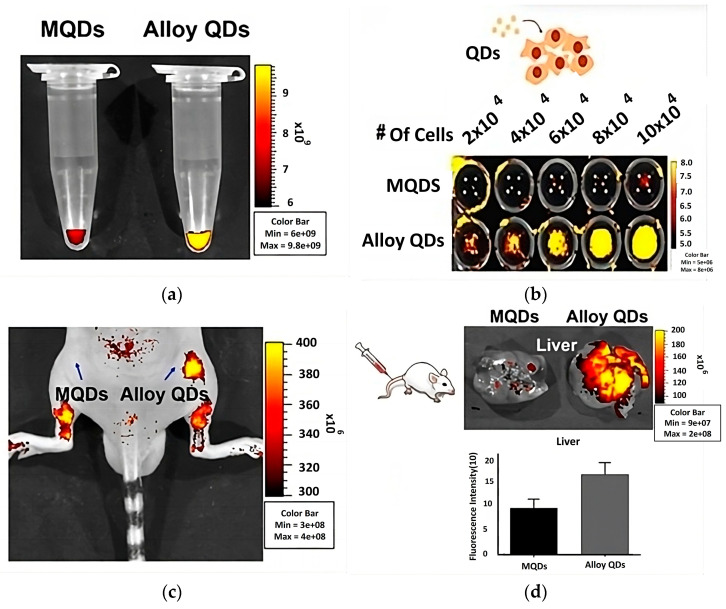
Fluorescence microscopy images of multi-layer Q.D.s (MQDs) and alloyed Q.D.s a) in: (**a**) microtube; (**b**) after bio-imaging of Hell cells; (**c**) after immediate mouse injection; (**d**) after one hour of mouse injection [15].

In 2022, a spectacularly high quantum yield of up to ~98% was obtained for quaternary CdSeZnS QDs, and it maintains its high value even after surface modification with a slight decrease (~84.7%), which is improved compared to the binary QDs where the QY decreased from ~84 to ~56%. These quantum dots were used as CdSeZnS/ZnS core/shell for KB cells (Hella cells) targeted imaging and compared to the binary multi-layer quantum dots MQDs as illustrated in Figure 21. of fluorescence microscopy (in vivo), where the strong fluorescence signal of the quaternary alloyed sample can be noticed over the multi-layer binary QDs inside the microtube (Figure 21a) and after interaction with KB cells, where the signals become stronger with increasing cell concentration (Figure 21b). After injecting the mice with the Hella cells treated with MQDs and alloyed QDs (Figure 21c), the enhancement of the fluorescence intensity of the cells treated with alloyed QDs was remarkable after one hour of injection (Figure 21d) [15].

## 5. Conclusions and Future Outlook

This review provides a detailed overview of alloyed (ternary and quaternary) cadmium-based quantum dots, their synthesis, properties, and applications. Through this article, we have discussed the most common preparation techniques for this species of nanocrystals, including hot injection, which is a widespread method to obtain quantum dots owing to different advantages. However, this technique has some drawbacks. In addition, co-precipitation, successive ionic layer adsorption and reaction (SILAR), hydrothermal, and microwave-assisted preparation techniques are discussed in the same manner, demonstrating the required conditions and environment for each method.

Alloyed cadmium chalcogenides have unique optical and structural properties, like their high PL quantum yield of up to 98% and their wide absorption band. The most attractive feature of these properties is that they can be manipulated by controlling the compositional ratio even when their size is kept constant. Composition is one of the main factors that can alter the alloyed Q.D.’s properties; there are other factors like time of reaction, temperature doping, and passivation. 

The properties of cadmium-based quantum dots have led to great development in many applications where these quantum dots represent the foundation stone that improves the quality of devices like solar cells, light-emitting diodes, and biosensors. 

Alloyed quantum dots are strong research subjects that may drive the wheel of many innovations; with the increasing demand for these Q.D.s, some aspects must be taken into consideration for future work:

1. Applying more research to strictly determine the best synthesis conditions to maintain high PL quantum yield and stability even without passivation;
2. To put a lot of effort into understanding what type of electrical transition may occur in these quantum dots, investigate the different scenarios for these transitions that govern the quantum dot properties;
3. To carry out synthesis techniques for high production.

We believe that alloyed Cadmium chalcogenide Q.D.s are promising nanomaterials. In particular, quaternary Q.D.s can have great potential once their properties are controlled since they present strong research tools that may lead to outstanding applications.

## Figures and Tables

**Table 1:** Advantages and disadvantages of alloyed QDs synthesis techniques.

Synthesis Technique	Advantages	Disadvantages	Ref.
Hot-injection	narrow particle size distribution	complicated preparation	[57,58,59,60,61]
	cost effective	not—applicable for large-area manufacturing	
		low repeatability	
Co-precipitation	facile	long time preparation	[51]
	fast		
	ability to manage size and composition depending on reaction conditions		
	applicable for large-area manufacturing		
	energy saving		
SILAR	facile	long time preparation	[45,62,63]
	no need for a vacuum or high temperature		
	cost effective		
	applicable for large-area manufacturing		
Hydrothermal	facile	long time preparation	[51,64]
	ability to manage size depending on reaction conditions	hard to observe the reaction	
	energy saving	needs an expensive autoclave	
Microwave-assisted	facile	hard to observe the reaction	[53,54,65,66]
	fast		
	cost effective		
	ability to manage the volumetric and internal heating of reagents		
	time saving		

**Table 2:** Structural properties of alloyed Q.D.s.

Quantum Dot	Diffraction Planes	Crystal Structure	Ref.
CdSeS	(111), (220), (311)	zinc blend	[38]
CdSeS	(111), (200), (311)	zinc blend	[19]
CdSSe	(100), (110)	hexagonal	[10]
CdSeTe	(111), (220), (311)	cubic zinc blend	[34]
CdSeTe	(111), (220), (311)	zinc blend	[33]
CdTeSe	(111), (220), (311)	F43m zinc blend	[76]
CdSeTeS		zinc blend	[27]
CdZnSe		zinc blend	[77]
CdZnSeS	(111), (220), (311)	zinc blend	[78]
CdZnSeS	(111), (220), (311)	zinc blend	[26]

**Table 3:** Photovoltaic parameters for different QDSSCs.

Photoanode	V_oc_ (V)	J_sc_ (A cm^−2^)	F.F. (%)	η (%)	Ref
CdSeTe	0.670	3.28	42.3	0.93	[34]
Cu_0.1_CdSeTe	0.586	2.62	53.1	1.13	
Cd_0.75_Zn_0.25_S	0.725	11.66	42.8	3.62	[46]
Cd_0.25_Zn_0.75_S	0.795	3.06	8.6	0.21	
CdSeTe (Au-CE)	0.592	1.930	54.276	0.758	[83]
CdSeTe (Pt-CE)	0.730	2.630	60.664	1.174	

## Data Availability

No new data was created.
